# Does movement influence representations of time and space?

**DOI:** 10.1371/journal.pone.0175192

**Published:** 2017-04-04

**Authors:** Jonna Loeffler, Markus Raab, Rouwen Cañal-Bruland

**Affiliations:** 1 Institute of Psychology, German Sport University Cologne, Cologne, Germany; 2 School of Applied Sciences, London South Bank University, London, United Kingdom; 3 Institute of Sport Science, Friedrich-Schiller-Universität Jena, Jena, Germany; University of Melbourne, AUSTRALIA

## Abstract

Embodied cognition posits that abstract conceptual knowledge such as mental representations of time and space are at least partially grounded in sensorimotor experiences. If true, then the execution of whole-body movements should result in modulations of temporal and spatial reference frames. To scrutinize this hypothesis, in two experiments participants either walked forward, backward or stood on a treadmill and responded either to an ambiguous temporal question (Experiment 1) or an ambiguous spatial question (Experiment 2) at the end of the walking manipulation. Results confirmed the ambiguousness of the questions in the control condition. Nevertheless, despite large power, walking forward or backward did not influence the answers or response times to the temporal (Experiment 1) or spatial (Experiment 2) question. A follow-up Experiment 3 indicated that this is also true for walking actively (or passively) in free space (as opposed to a treadmill). We explore possible reasons for the null-finding as concerns the modulation of temporal and spatial reference frames by movements and we critically discuss the methodological and theoretical implications.

## Introduction

“Space, motion, and time cannot be elucidated by discovering an 'inner' layer of experience in which their multiplicity is erased and really abolished. For if this happens, neither space, nor movement, nor time remains…” [1].

What seemed clear to the French philosopher Merleau-Ponty already in 1945 [1], is surprisingly often neglected in psychological research about temporal and spatial representations in humans: Our knowledge of the mechanisms underlying temporal and spatial representations is incomplete without understanding their relation with movement. Indeed, the majority of studies in different research areas such as neurosciences, linguistics, and cognition (for overviews from different perspectives see e.g., [2,3]) focus on the relatedness between time and space, but often do not consider the influential role of movement. To this end, the current study aims at examining the impact of movements on the construction of temporal and spatial representations (note that we use the terms ‘representation’ and ‘concepts’ interchangeably, both referring to ‘conceptual representations’; see [4]).

In this context, the conceptual metaphor theory [5] provides a pertinent theoretical backdrop as it describes the functional relationship between abstract concepts such as those of time and space and sensorimotor experiences. In specific, Lakoff and Johnson [5] argue that physical experiences with the environment form the foundation for the generation of abstract conceptual knowledge. In research on embodied cognition this is sometimes referred to as the grounding hypothesis [6,7]. A second argument put forth by Lakoff and Johnson [5] is that abstract (e.g. temporal) concepts are expressed by metaphors from more concrete (e.g. spatial) conceptual domains. Spatial metaphors used to describe temporal events are, for instance, “travelling in time”, “as time flies by”, or “the worst is behind”.

An influential study by Boroditsky [8] provided compelling evidence for shared conceptual structures of temporal and spatial representations. Her findings suggested that spatial conceptual knowledge is used to represent events in time, but temporal conceptual knowledge is not (or to a lesser extent, see e.g., [9,10]) used to represent events in the spatial domain. But how do we conceptually “organize” events in time and space?

Clark [11] advocated two frames of reference that people use when they refer to time and space, namely an ego-moving frame of reference and an object-moving frame of reference. In the ego-moving reference frame, a person perceives and describes the world from an ego perspective, that is, the ego moves along the time-line in the direction of the future. “I am approaching the end of the bridge” serves as an example for an ego-moving reference frame in the spatial domain; “I am approaching the end of my holidays” provides for an example of the temporal domain. In contrast, in an object-moving frame of reference, one perceives and describes the world from a rather passive perspective, in which objects and time approach an otherwise stationary observer. “The church will pass by before the bridge” serves as an example for an object-moving reference frame in the spatial domain; and “Christmas will pass by before Easter” provides for an example of an object-moving reference frame in the temporal domain [8].

**Fig 1:**
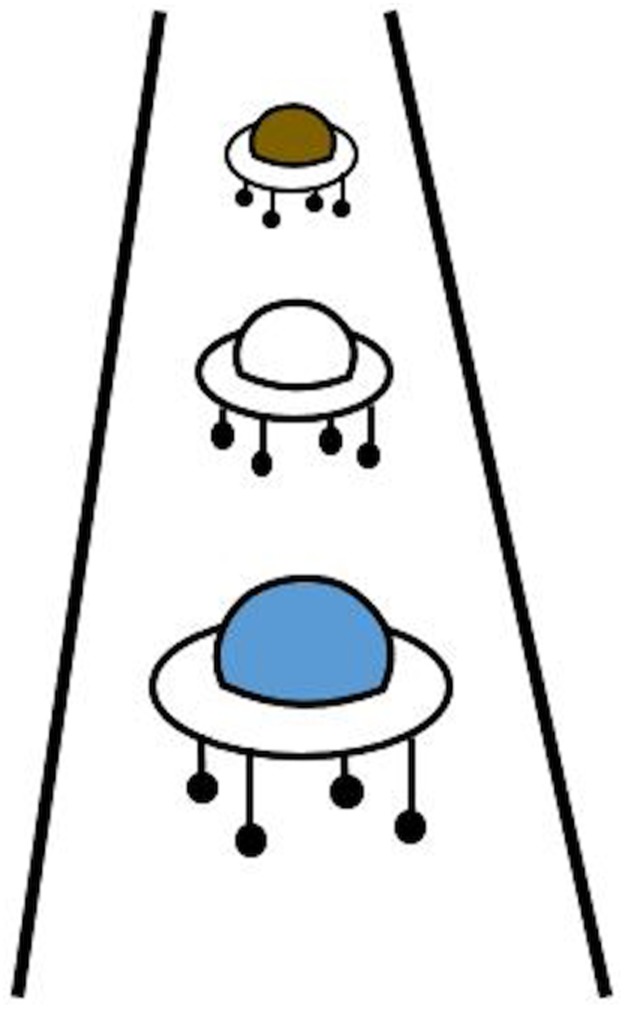
The ambiguous spatial target. Participants were invited to answer the question: “Which one of these widgets is ahead?” (picture adapted from Boroditsky [8]) by naming a color. The answer “blue” (near) is congruent to an ego-moving reference frame, whereas the answer “brown” (far) is congruent to an object-moving reference frame.

An elegant, experimental way to scrutinize Clark’s two frames of reference proposal [11] is to confront participants with questions and metaphors that are ambiguous as concerns their temporal and spatial connotations. For instance, one well-researched ambiguous question in the temporal domain is the ‘Wednesday’s meeting question’ [12], in which participants are invited to answer the following question: “Next Wednesday’s meeting has been moved forward two days. What day is the meeting now that is has been rescheduled?” [8,13,14]. The question was first applied by McGlone and Harding [12], and has since been validated and replicated numerous times (e.g., [15,16]). For an example on how to examine Clark’s two frames of reference proposal for the spatial domain, please consult Fig 1 (and its figure caption for an explanation).

As a case in point, the underlying experimental idea in Boroditsky [8] was to either induce an ego-moving or object-moving frame of reference and to test the primed reference frame’s effect on the response to the ambiguous question. Since then, several studies have used this paradigm and conceptually replicated the main finding that representations of time are modulated by different motion primes. These studies used various manipulations, including mental imagery [17], abstract motion primes (e.g., counting in one direction or filling letters; [18], observing moving objects on a screen [19], or sentences with motion words [20]).

Notably, however, the majority of studies thus far applied metaphoric (and therefore abstract) manipulations that may (or may not) have—advertently or inadvertently—activated sensorimotor experiences. In an attempt to activate sensorimotor experiences, Boroditsky, Ramscar, and Frank [21] were actually the first to examine whether actively moving in space would induce an ego-moving frame of reference, and pulling an object towards oneself would induce an object-moving frame of reference. In particular, in one of their experiments participants had to either ride (ego-moving condition) or rope (object-moving condition) an office chair. In the riding condition, participants sat on and moved a chair with their legs towards a particular target; in the rope condition, participants held a rope fixed to the chair and pulled it towards them. Results showed no different responses to the Wednesday-meeting question depending on the movement condition. That is, inducing ego- and object-moving frames of reference by means of movements did not differentially modulate representations of time. Why did the imagination of a movement result in a modulation of a temporal concept, whereas the execution of a real movement did not result in a modulation of a temporal concept?

The findings above are not necessarily in line with some of the main tenets of the conceptual metaphor theory [5] and embodied cognition theories [6,7], thus an answer to the question requires closer inspection. From an embodied cognition perspective [6,7] physical experience (like walking forward or backward) form the foundation of abstract conceptual knowledge. Spatial concepts like “forward” and “backward” are strongly associated with forward and backward movements. Based on the conceptual metaphor theory, temporal concepts like “future” and “past” are built on these spatial, more concrete concepts. Associations between movements and spatial or temporal concepts have been shown to emerge directly, that is, within brief periods of time (e.g., [21–23]). Therefore, it ought to be expected that the on-line execution of forward and backward movements should cause modulations of temporal and spatial reference frames.

In support of the close link between movement, time, and space, the direction of body motion has been shown to mediate directional shifts of spatial attention [22] and voluntary movements have been shown to modulate time perception [23]. In addition, an altered experience of time and space has been shown to involve theta activity in regions that are related to a sense of the body [24]. Further evidence for this coupling stems from recent research on embodied cognition, showing that moving forward is associated with thinking about the future, and moving backward with thinking about the past [25–30]. Together, these studies seem to indicate a specific, directional influence of movements on temporal and spatial representations. In the remainder of the article, we refer to this as the *movement direction-dependent hypothesis*.

Alternatively, it is also conceivable that movements, independent of the presence and role of objects in the environment and despite being executed in opposite directions (e.g., walking backward vs. moving forward), might be generally perceived from an ego-moving frame of reference and as being future-directed. This is, first, because the agent is executing a self-induced and self-controlled movement thereby enhancing the feeling of being an active agent, and second, because movements as they unfold are—per definition—always future-oriented [31] and may hence activate future-related temporal representations [32]. If true, then otherwise specific and directional effects of moving either forward or backward on temporal representations may be overridden, and rather unselectively activate future-oriented associations and concepts. We refer to this as the *movement direction-independent hypothesis*.

To test these hypotheses and scrutinize whether changes to the motor system (i.e., by means of whole-body movements) impact on perceptions of time and space, in the current study we asked participants to walk forward or backward or to stand still (control condition) on a treadmill and to answer the either *temporally* ambiguous Wednesday-meeting question (Experiment 1) or a *spatially* ambiguous question (Experiment 2, see Fig 1). To avoid carry-over effects from the temporal to the spatial question and vice versa [17], we used a between-subject design. As dependent variables the answers and response times to the temporal and spatial questions were measured. We measured response times, as it is conceivable that on-line movement as a prime may not be strong enough to change the answer to the ambiguous spatial and temporal questions, but might affect the answers in form of an incongruence effect, which would be observable in slower response times of, e.g. participants that walk forward and answer “Monday”.

In general, we aimed to conceptually replicate the finding that motion primes modulate representations of time and space, by applying self-generated motion instead of abstract motion. We predicted larger effects on responses in the temporal domain (Experiment 1), than on responses in the spatial domain (Experiment 2) as temporal representations have been shown to be more variable (or less stable) than spatial representations [8,30,33,34]. Further, for both Experiment 1 and Experiment 2 we tested the *movement direction-dependent hypothesis* against the *movement direction-independent hypothesis*. The *movement direction-dependent hypothesis* predicted that walking forward would result in more Friday answers in Experiment 1 (temporal domain) and more ‘the widget is further away’ answers in Experiment 2 (spatial domain, see Fig 1), whereas walking backward would result in more Monday answers in Experiment 1 and ‘the widget is closer by’ answers in Experiment 2. The *movement direction-independent hypothesis* differed from the *movement direction-dependent hypothesis* in that it predicted more Friday answers in Experiment 1 and more ‘widget is further away’ answers in Experiment 2 independent of whether participants walked forward or backward. In a follow-up investigation, we aimed to rule out a potential confound in Experiment 1 and thereby asked participants to walk actively forward *in space* (as opposed to walking on a treadmill, follow-up Experiment 3).

## Experiment 1

In Experiment 1, we examined the *movement direction-independent hypothesis* against the *movement direction-dependent hypothesis* in the temporal domain. To this end, participants were asked to answer an ambiguous temporal question while they walked forward, backward or stood on a treadmill.

### Method

#### Participants

An a priori Gpower analysis with an estimated effect size of *w* = .23 (based on a first testing period, *N* = 63), an alpha = .05 and a recommended power = 0.8 [35] revealed a required sample size of at least 183 participants (61 per group). [In a first testing period we assumed a larger effect size of .54, based on the smallest effect size (Experiment 2) in Matlock et al. [18]. The data based on the first testing period revealed a tendency towards one of the hypotheses. To ensure enough power and to be sure about the effect, we extended the first testing period of Experiment 1 and adjusted the assumed effect size.] Therefore, we tested 211 participants (109 female) in total. The mean age of the participants was 28.15 (*SD* = 12.06) years. Primary inclusion criteria for the participants were sufficient knowledge of English, age between 18 and 65, and no health restrictions with regard to their walking abilities. All participants provided written informed consent and were free to withdraw from testing at any time. The experiment was approved by the ethical committee of the German Sport University Cologne.

#### Apparatus and stimulus

The ambiguous question (Wednesday-meeting question from Boroditsky [8]) was presented auditorily and the answer (either “Friday” or “Monday”) was given verbally. For this purpose, the Wednesday-meeting question was recorded from a native English speaker. Due to the fact that the Wednesday-meeting question is not ambiguous in German [13] we did not translate the question into German but stayed with the English version. A pilot study confirmed (chi-square test with an expected frequency of 50% of each answer) that the ambiguity of the Wednesday-meeting question remained, when native German speakers were asked in English, χ^2^(1, *N* = 29) = .86, *p* = .35.

The question was presented via a wireless headset (Sennheiser MB Pro 2UC). The method of presenting the stimulus auditorily and recording vocally produced answers had the advantage that any reference to a spatial relation (e.g., when lifting the arm to point or press a button) was omitted. The target was presented on-line after four minutes (to ensure that the walking manipulation was not affected by the participants’ walking right before entering the treadmill), in real-time during body motion, meanwhile participants kept walking forward or backward (or standing) with a speed of 3 km/h (normal walking speed, examined during pilot work) on a standard treadmill. A sociodemographic questionnaire was administered using SoSci Survey [36] and completed by the participants after the experiment. The questionnaire included relevant sociodemographic questions as well as a self-assessment of the participants’ English abilities.

#### Procedure

After participants had provided written informed consent and had been randomly assigned to one of the three different conditions (between-subject design: walking forward, walking backward, standing) they got a standardized instruction in English, according to their assigned condition. Two of the groups were subjected to a movement manipulation. Participants in these two groups either were instructed to walk forward or to walk backward on a treadmill for four minutes and to then answer the auditorily presented question as fast as possible. The control group was not subjected to any kind of movement, but was asked to rest (i.e., stand still) for four minutes. In order to account for familiarity differences between forward (more familiar) and backward (less familiar) walking, participants assigned to the backward condition received the opportunity to familiarize themselves with backward walking. None of the participants in the backward condition requested more than half a minute for familiarization. After reading the instruction, participants put on wireless headphones and stepped onto the treadmill for three minutes. After responding to the ambiguous temporal question, participants left the treadmill and filled out the sociodemographic questionnaire on a laptop. After completing the questionnaire participants were debriefed individually.

#### Data analysis

Of the 211 participants that were tested, 13 were excluded due to an answer that was neither “Monday” nor “Friday” or self-reported “poor” English abilities. The final sample for the analysis was therefore *N* = 198.

Auditory analysis of the verbal responses was performed with Audacity [37]. Each file was analyzed individually with a standardized procedure (Audacity sound finder parameters: treat audio below this level as silence: 35 dB; minimum duration of silence between sounds: 0.1 s; label starting point: 0.001 s). Response times were extracted by subtracting stimulus offset from answer onset.

Statistical analysis was performed with RStudio Team [38]. To analyze the answers, a chi-square test was conducted (forward, backward, control). Cramer’s V was calculated as an effect size estimator and is reported only for *χ*^2^ > 1. To quantify how much the data should shift our belief in favor of the null or the alternative hypothesis, we calculated the independent multinomial Bayes Factor for contingency tables (BF_01_, where 1 means that they are equally likely, larger values indicate more evidence for the null hypothesis, and smaller values indicate more evidence for the alternative, calculated with RStudio Team [38]).

To analyze the response times, a 3 x 2 analysis of variance (ANOVA) was performed. The between-subject factors were condition (forward, backward, control) and answer (Monday, Friday). As we had two a priori hypotheses (movement direction-independence or movement direction-dependence) we set two a priori contrasts (Movement vs Standing, Forward vs Backward). Further in the analyses of the response times we calculated the Bayes Factor (JZS, calculated with RStudio Team [38]) for the full model and for both a priori contrasts (Movement vs Standing, Forward vs Backward). To find out if the model with or without the interaction explains the data for each contrast better we compared the model with and without the interaction term [39].

Participants that reported to have 1 = “poor” English skills (scale ranging from 0 = “I don’t speak English” to 6 = “mother tongue”) were excluded from the analysis of the response times in all Experiments. In most of the cases, these participants were identical to those who provided a wrong answer. To analyze the relationship between self-reported English abilities and the answer to the Wednesday-Meeting question, we calculated the Bayes Factor for contingency tables (BF_01_). To analyze the relationship between self-reported English abilities and the response time to the Wednesday-Meeting question, we calculated the Bayes Factor for correlation pairs (BF_01_).

Outlier correction performed by winsorizing outlying observations [40] included 14% outliers. Winsorizing did not change the overall pattern of the results. Assumptions were checked by plotting Cooks’ Distance and the histograms of the studentized residuals [38]. Cooks’ Distance was below 1 for all observations, and the distribution of the studentized residuals showed that the deviation of the residuals was normally distributed for all models. Effect sizes were calculated as eta-squared values (η^2^) and are reported only for *F* > 1. The significance criterion for all analyses was *p* = .05.

### Results and discussion

#### Answers

**Fig 2:**
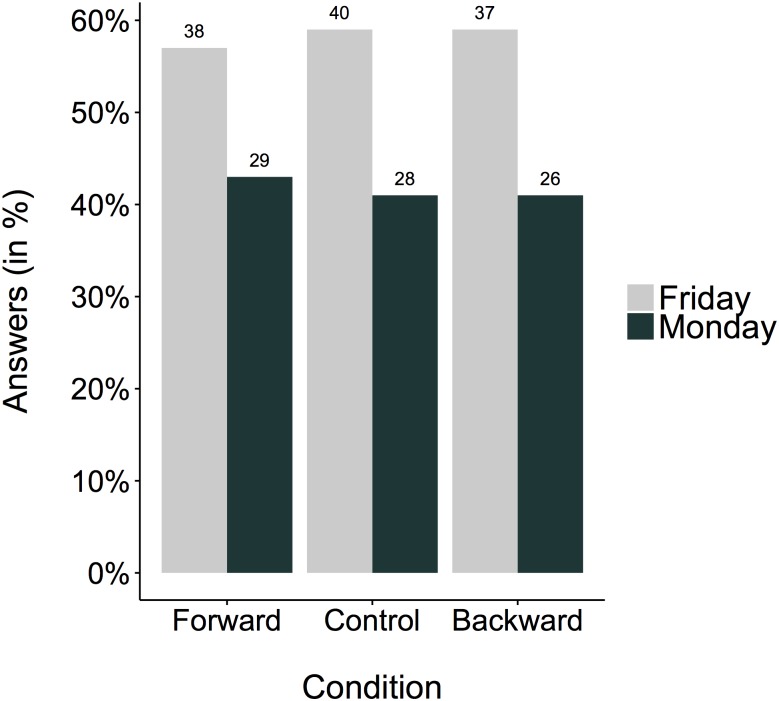
Percentage of Friday and Monday answers plotted for the three different groups (i.e., walking conditions). The total number of answers is printed above each bar.

We examined whether whole-body movements influence responses to the Wednesday-meeting question. The answer is no, they did not. For a summary of the results, see Fig 2. On a descriptive level, the proportion of “Friday” and “Monday” answers was essentially the same in all conditions. On a statistical level, this observation was confirmed χ^2^(2, *N* = 198) = .08, *p* = .96. The corresponding independent multinomial Bayes Factor is equal 24.68, indicating that the data provide 24.68 times more evidence for the null hypothesis of independence than for the alternative hypothesis of dependence, speaking in favor of the null hypothesis that movement does not influence the answer to the ambiguous temporal question. The control group confirmed the result of the pilot test and did not significantly differ from a 50/50 expectancy, χ^2^(1, *N* = 68) = 2.12, *p* = .15, *w* = .18. The analysis of the control variable English abilities indicated that the Bayes Factor against the null hypothesis was BF_01_ = 2.04, speaking in favor of the null hypothesis (= self-reported English abilities did not affect the answers). The analysis of the control variable age revealed no significant relation to the answer to the Wednesday-Meeting question.

#### Response times

**Fig 3:**
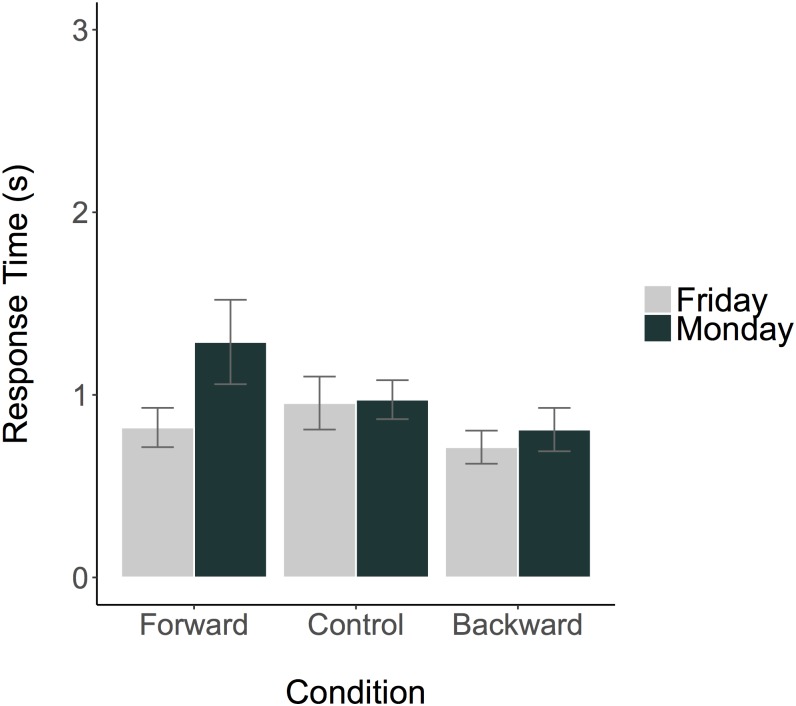
Mean response times (winsorized) to the ambiguous temporal question per condition and answer. Error bars represent standard errors.

Response times per answer and condition are plotted in Fig 3. The ANOVA (Answer x Condition) revealed that none of the main effects, contrasts, or interactions significantly influenced the response times. Therefore, none of the a priori hypothesis could be confirmed: The results indicated no interaction between the first contrast (Forward vs Backward) and Answer, nor an interaction between the second contrast (Moving vs Standing) and Answer. We further calculated and compared the Bayes Factors [39] of the full model and of both a priori hypotheses separately. The Bayes Factor for the full model (including both main effects Answer and Condition and their interaction) against the intercept-only null hypothesis was BF_01_ = 12.72, speaking in favor of the null hypothesis. As we were mainly interested in the a priori contrasts and the respective interactions, we compared the models with the a priori contrasts (Forward vs Backward, Moving vs Standing) each with and without the interaction term. For the contrast testing the movement direction-dependent hypothesis, there was a small preference for the model without the interaction term over the model with it (BF_01_ = 2.0); the same was true for the contrast testing the movement direction-independent hypothesis (BF_01_ = 2.19). The analysis of the control variable self-reported English abilities indicated that the Bayes Factor against the null hypothesis was BF_01_ = 2.02, speaking in favor of the null hypothesis (= self-reported English abilities did not affect the response time). The analysis of the control variable age revealed no significant relation to the response times.

In sum, walking forward or backward did not modulate the temporal reference frame, reflected by the answer to the ambiguous temporal question. Numerically, response times for participants who walked forward and answered “Monday” were longer than those who walked backward or stood and answered “Monday”. However, this was not confirmed statistically by the ANOVA, and also the Bayesian Statistic indicated that the data provide about 12 times more evidence for the null hypothesis than for the alternative hypothesis.

## Experiment 2

The results of Experiment 1 indicate that whole-body movements do not impact on temporal reference frames. Experiment 2 addressed the question whether and how movement impacts spatial representations. To this end, in Experiment 2 participants were asked to answer an ambiguous spatial question while they walked forward, backward, or stood still on a treadmill.

### Method

#### Participants

Due to a lack of studies that report a comparable priming of the Widget-question, we calculated the sample size assuming a slightly smaller effect than for the temporal target in Experiment 1. As both Experiments were run in parallel, we took Matlock et al. [18] as reference instead of the effect size of the extended Experiment 1. The a priori Gpower analysis with an estimated effect size of w = .45, an alpha = .05 and a recommended power = 0.8 [35] revealed a required group size of at least 39 participants per group.

We therefore invited 120 participants (40 female) to take part in the experiment. The mean age of the participants was 24.1 (*SD* = 6.8) years. Primary inclusion criteria for the participants were sufficient knowledge of English, age between 18 and 65, and no health restrictions with regard to their walking abilities. None of the participants had participated in Experiment 1. All participants provided informed consent and were free to withdraw from testing at any time. The experiment was approved by the ethical committee of the German Sport University Cologne.

#### Stimulus and apparatus

The general set-up including the treadmill walking manipulation and design was identical to Experiment 1. However, after four minutes of walking (or standing), an ambiguous *spatial* question was presented, meanwhile participants continued walking. A pilot study confirmed (chi-square test with an expected frequency of 50% of each answer) that the ambiguity of the Widget-question remained, χ^2^(1, *N* = 31) = .81, *p* = .37, when native German speakers were asked in English.

The spatial ambiguous question “Which one of these widgets is ahead?” (Fig 1, adapted from Boroditsky [8]) was presented via a wireless headset (Sennheiser MB Pro 2UC) four minutes after the start of the treadmill manipulation. Simultaneously, a picture (Fig 1) of three vertically aligned objects appeared on the screen of video-glasses (Virtual Private Theater System 4GB, 72 inch). The illustrated widgets were colored (blue, brown). The color was counterbalanced, to exclude any possible color effects on response times. The first phoneme was identical for each of the colors to provide the possibility to detect a clear word onset. Although the participants answered by naming a color, we refer to the answers in the remainder of the article with their spatial characteristics “near” and “far”, for a color-independent description.

#### Procedure

The general procedure was the same as in Experiment 1, with the only difference being the presentation of the spatial ambiguous question, which included a picture (Fig 1) that was presented on video-glasses, and the corresponding auditorily presented question. Participants had to answer as quickly as possible by naming the color of the widget. The procedure after answering the question was the same as in Experiment 1.

#### Data analysis

Of the 120 participants that were tested, nine were excluded due to a misunderstanding of the question (e.g., providing the answer “white”) or self-reported “poor” English abilities. The final sample for the analysis was therefore *N* = 111. Auditory and statistical analyses were identical to Experiment 1. Outlier correction performed by winsorizing outlying observations [40] included 9.9% outliers.

### Results and discussion

#### Answers

**Fig 4:**
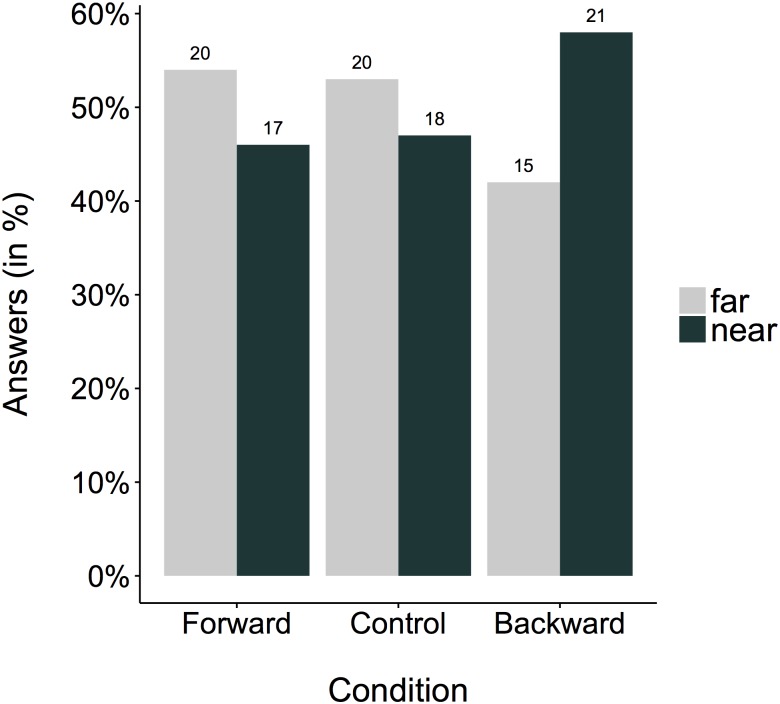
Percentage of “far” and “near” answers to the ambiguous spatial question plotted for the three different groups (i.e., walking conditions). The total number of answers is printed above each bar.

We examined whether whole-body movements influence responses to the Widget-question. The answer is no, they did not. For a summary of the results, see Fig 4. Forward as well as backward whole-body movements produced almost an equal amount of “far” responses (forward: 46%; backward: 58%) when compared to the control group (47%). The control group χ^2^(1, *N* = 38) = .11, *p* = .76 confirmed the result of the pilot test and did not significantly differ from a 50/50 expectancy. As in Experiment 1, the answer was not influenced by the movement direction χ^2^(1, *N* = 111) = .34, *p* = .5. The analysis of the control variable English abilities indicated that the Bayes Factor against the null hypothesis was BF_01_ = 4.74, speaking in favor of the null hypothesis (= self-reported English abilities did not affect the answers). The analysis of the control variable age revealed no significant relation to the answer to the Widget-question.

#### Response times

**Fig 5:**
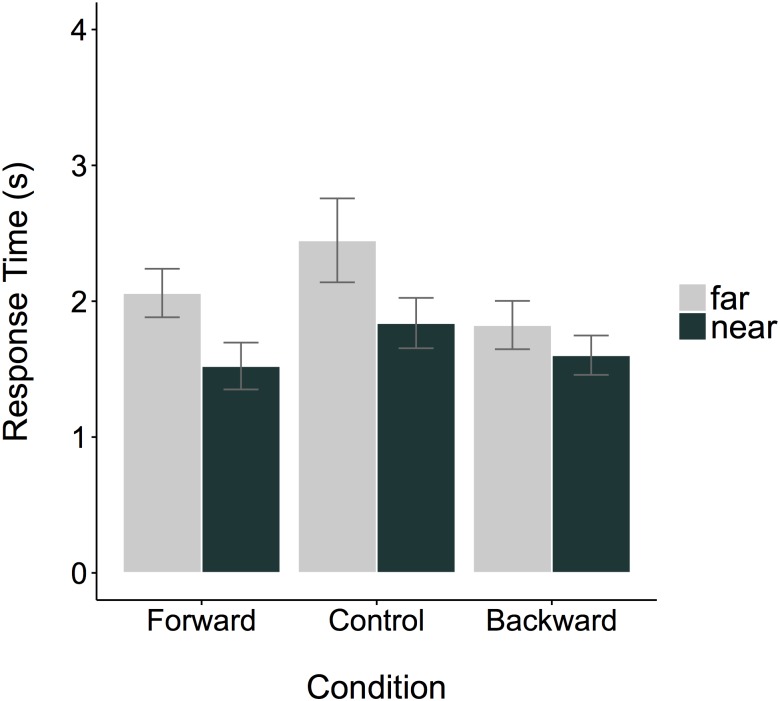
Mean response times (winsorized) to the ambiguous spatial question per condition and answer. Error bars represent standard errors.

Response times per condition and answer are plotted in Fig 5. The ANOVA (Answer x Condition) revealed a main effect of Answer, *F*(1, 95) = 7.35, *p* = .007, *η*^*2*^ = .06. Participants that answered “near” had shorter response times than participants that answered “far”. The planned contrast Forward vs Backward, *F*(1, 95) = 4.9, *p* = .03. *η*^*2*^ = .04 was significant. Participants that walked forward answered slower than participants that walked backwards. Neither the interaction between the first contrast (Forward vs Backward) and Answer *F*(1, 95) = .84, *p* = .36, nor the interaction between the second contrast (Moving vs Standing) and Answer *F*(1, 95) = .74, *p* = .11 were significant. We further calculated and compared the Bayes Factors [41] for the full model and for both a priori hypothesis separately. The Bayes Factor for the full model (including both main effects Answer and Condition and their interaction) against only the intercept-only null hypothesis was BF_01_ = 1.14, meaning that the data are explained by the intercept-only null hypothesis as well as by the alternative model. As we were mainly interested in the interaction, we analyzed whether the data are explained better by the model with or without the interaction term. The resulting Bayes Factor was BF_01_ = 4.77, speaking in favor of the model without the interaction term. Because we were mainly interested in the a priori contrasts and the respective interactions, we compared the models with the a priori contrasts (Forward vs Backward, Moving vs Standing) each with and without the interaction term. Results revealed for both a priori contrasts a comparable preference of the model without over the model with the interaction term (movement direction-dependent: BF_01_ = 3.04, movement direction-independent: BF_01_ = 2.18). The analysis of the control variable self-reported English abilities indicated that the Bayes Factor against the null hypothesis was BF_01_ = 7.45, speaking in favor of the null hypothesis (= self-reported English abilities did not affect the response time). The analysis of the control variable age revealed no significant relation to the response times.

In sum, answers to the ambiguous spatial task did not yield differences among conditions. This means that whole body movements did not change the frame of reference used to answer the spatial ambiguous question nor did they impact the response times. Consequently neither the *movement direction-independent hypothesis* nor the *movement direction-dependent hypothesis* could be confirmed for the spatial domain.

## Discussion Experiment 1 and 2

To summarize, in Experiments 1 and 2 movement (i.e., walking) influenced neither the answer to the ambiguous temporal question (Experiment 1) nor the answer to the ambiguous spatial question (Experiment 2). Measurement of the response times was applied to control for general differences of difficulty in moving (forward, backward) vs standing condition and to compare both hypotheses directly. The results showed no significant interaction, and the Bayesian Statistic confirmed for both Experiments 1 and 2 that the data showed more evidence for the null hypothesis than for the alternative hypothesis.

Yet, if our theoretical reasoning was sound, then why did we not find an influence of movement on the temporal and spatial reference frame? One potential issue to consider is our moving manipulation. As the aim of the study was to prime abstract reference frames by means of concrete whole-body movements, the conceptual setting of the movement manipulation should reflect those of the reference frames (ego-moving, object-moving). It could be argued that the reference frames were not perfectly reflected in the present design, because participants did not really move forward in space while executing a forward walking movement on a treadmill. On the one hand, to activate an ego-moving reference frame, it might be necessary to execute a forward walking movement that leads to a forward movement in space. On the other hand, if somebody walks forward in space the visual input ‘flies by’, which would be analogous to an object-moving reference frame. To control for different visual input between the walking conditions and to keep the walking speed equal for all participants, we had decided to apply the walking condition on the treadmill for Experiment 1 and 2. Nevertheless, to rule out that moving forward in space and the corresponding change in visual input did not account for our null-findings, we ran an additional experiment.

## Experiment 3

The results of Experiment 1 and 2 indicate that whole-body movements do not impact on temporal and spatial reference frames. To investigate if the lack of evidence for a modulation of temporal reference frames in Experiment 1 and 2 is caused by the special characteristics of the movement manipulation (i.e. participants did not walk forward in space, but walked on a treadmill), we conducted an additional experiment: In a first condition, participants walked forward in space (active ego-moving condition). In a second condition, participants sat still and saw an office-chair approaching them (object-moving condition). In a third condition, participants sat on an office-chair and were passively moved forward (passive ego-moving condition; for a similar approach see Boroditsky and Ramscar [42]). The dependent variable was the answer to the Wednesday-Meeting question (same as in Experiment 1).

Our hypotheses for Experiment 3 were two-fold: If movement is indeed independent of the temporal reference frame (reflected by the answer to the Wednesday-Meeting question), it should not make a difference if participants generate a forward walking movement on a treadmill or in real space. If the movement *in space* is able to activate an ego-moving reference frame, actively moving forward in space should activate an ego-moving reference frame, resulting in more “Friday” than “Monday” answers than in the object-moving condition. According to embodied cognition studies comparing the impact of active and passive movements on abstract processing, the effect should be larger in the active than in the passive ego-moving condition (e.g., [43]).

Our hypothesis with respect to the object-moving condition was that sitting still and seeing something approaching should activate an object-moving reference frame and hence result in more “Monday” answers than “Friday” answers.

### Method

#### Participants

Because in Experiment 3 we were primarily interested in the modulation of the reference frames, we calculated the sample size for a Chi-square test (instead of an ANOVA, as in Experiments 1 and 2), taking a slightly smaller effect size than the smallest effect size reported in Matlock et al. [18] as reference (same as in Experiment 2). The a priori Gpower analysis with an estimated effect size of w = .45 [18] an alpha = .05 and a recommended power = 0.8 [35] revealed a required sample size of at least 48 participants. We invited 90 participants (50 female) to take part in the experiment.

The mean age of the participants was 22.58 (*SD* = 3.38) years. Primary inclusion criteria for the participants were sufficient knowledge of English, age between 18 and 65, and no health restrictions with regard to their walking abilities. None of the participants had participated in Experiment 1 or 2. All participants provided informed consent and were free to withdraw from testing at any time. The experiment was approved by the ethical committee of the German Sport University Cologne.

#### Apparatus and stimulus

The ambiguous temporal question (Wednesday-meeting question from Boroditsky [8]) was presented auditorily and the answer (either “Friday” or “Monday”) was given verbally (same as in Experiment 1). A sociodemographic questionnaire was administered using SoSci Survey [36] and completed by the participants after the experiment. The questionnaire included relevant sociodemographic questions as well as a self-assessment of the participants’ English abilities.

#### Procedure

The movement tasks for the different conditions were as follows: In the active ego-moving condition participants walked from a starting-point to a marked finish line (distance between starting point and finish line: 15 m). In the passive ego-moving condition, participants sat on an office chair with casters, situated at the starting point. The experimenter stood behind the participant sitting in the office chair and walked with normal walking speed forward, meanwhile pushing the participant sitting in the chair constantly forward towards the finish line. In the passive object-moving condition participants sat on a chair at the starting point facing an empty office chair, which was standing at the finish line. They were instructed to watch the chair while the investigator pulled it with a rope towards the participant. The investigator was standing behind the participant, to prevent any influence of the investigators motor action on the participants’ judgments.

#### Data analysis

Of the 90 participants that were tested, 16 were excluded due to an answer that was neither “Monday” nor “Friday” or self-reported “poor” English abilities. The final sample for the analysis was therefore *N* = 74. The data were analyzed in the same way as in Experiment 1 and 2, with the only difference that, because in the present Experiment 3 we were primarily interested in the modulation of the reference frames, we only measured the answers provided and did not measure response times.

### Results and discussion

We examined whether moving actively or passively forward (compared to not moving at all) influences responses to the Wednesday-meeting question. The answer is no, it did not. On a descriptive level, the proportion of “Friday” and “Monday” answers was essentially the same in all conditions. On a statistical level, this observation was confirmed χ^2^(2, *N* = 74) = .1, *p* = .95. The corresponding independent multinomial Bayes Factor is equal to 9.51, indicating that the data provide around 9.51 times more evidence for the null hypothesis of independence than for the alternative hypothesis of dependence, speaking in favor of the null hypothesis that movement does not influence the answer to the ambiguous temporal question. The analysis of the control variable English abilities indicated that the Bayes Factor against the null hypothesis was BF_01_ = 31.15, speaking in favor of the null hypothesis (= self-reported English abilities did not affect the answers). The analysis of the control variable age revealed no significant relation to the answer to the Widget-question.

Contrary to the hypothesis that *actively moving forward in space*, *passively moving forward in space* versus *something approaching you* should result in different answer patterns to the ambiguous temporal question, the proportion of “Friday” and “Monday” answers showed no differences between conditions. The results add evidence that movement indeed does not activate a temporal ego-moving reference frame (reflected by the answer to the Wednesday-Meeting question), regardless of whether participants generate a forward walking movement on a treadmill (Experiment 1) or in real space (Experiment 3). Further, the results indicated that neither sitting still (Experiment 1) nor sitting still and watching something approaching (Experiment 3) activates an object-moving reference frame.

## General discussion

Together, the results of the present experiments seem to provide compelling evidence against the hypothesis that temporal and spatial reference frames are influenced by on-line whole-body movement. Although previous studies provided evidence that *abstract* movement activates temporal reference frames (e.g., forward or backward counting [18], but see also [13]), the present experiments indicate that the effect cannot be confirmed for *concrete* whole-body movement. Potential candidates to explain this unexpected finding are discussed in the remainder of the discussion.

First, it is conceivable that the English skills of our participants were not sufficient to understand the question. Therefore, our participants may have just guessed the answer to the temporal and spatial ambiguous question. However, we excluded participants that reported to have poor English skills or did not understand the question. Further, we did not find a relation between the (self reported) English skills and the answer provided or the response time in any of the experiments.

Second, methodological differences between former studies and the study at hand might explain the finding that whole-body movement did not change the answers to the ambiguous temporal and spatial questions. The main differences between former investigations of spatial and temporal reference frames (i.e., [4]) and the present investigation are that manipulation and target presentation occurred simultaneously (= instead of sequentially, e.g. [44]), and that a real walking movement was applied as an independent variable.

As concerns the former, the movement manipulation and the target presentation occurred simultaneously, based on the argument that just after having stopped a movement, another reference frame may be activated than when currently executing a movement. As a consequence of this on-line manipulation, we can rule out that the reason for our findings is contaminated by effects that occurred between the manipulation and the presentation of the target question, but we are able to directly link the results to the movement execution (see also [45]).

As concerns the latter, the execution of a walking movement was applied as independent variable. This is in contrast to former studies in which movement was applied in an indirect, abstract form (e.g., counting forward or backward, or imagining a movement, [18,42]). The unsuccessful attempt to test the same hypothesis with a method that varies in some way from previous studies (i.e., the execution of walking movements instead of imagining a movement) highlights the importance of conceptual replications [46]. Although temporal and spatial reference frames include different movement directions per definition, and the imagination of a directional movement primed one of the reference frames [45], the *execution* of directional movement modulated neither temporal nor spatial reference frames. Integrating our results into the literature about ambiguous temporal and spatial questions and the concept of different temporal and spatial reference frames, we can conclude that the answer to the ambiguous questions does not depend on self-generated movement (see also [21]). What does this result mean on a theoretical level?

We suggest that a proper characterization of the factors that modulate temporal and spatial representations will need to look beyond the information that comes from sensorimotor experience alone, but consider interactions between sensorimotor experience (e.g., [47]) culture (e.g., [13]) and language (e.g., [48–50]). Several studies have shown that language and culture are strongly influenced by sensorimotor experiences (for an overview see [51].) Further, language and culture are reported to modulate the reference frame that is used to answer ambiguous temporal or spatial questions (e.g., [13,48–50]). Therefore, it might be fruitful to examine interactions between sensorimotor experience, language, and culture and their weighted impact on temporal and spatial representations.

For example, a strong effect of language was reported by Bender et al. [13], without any additional effects of spatial primes or task order. That is, in Germany and China, the object-moving reference frame clearly predominated, whereas in the US, both the object-moving and ego-moving reference frame were adopted. The "young" Tongan participants revealed a pattern similar to that of the US participants, with preferences for the ego-moving as well as for the object-moving reference frame (as could be expected, because English is the official school language), whereas the "old" Tongan participants prefer the ego-moving reference frame for events in the future. Further, when measuring the preferred reference frame of participants in four different French-speaking countries, Hüther et al. [49] reported that although all four samples spoke the same language, they differed with respect to the extent the different reference frames were used. Nevertheless, animate (i.e., starfish and bird) and inanimate (i.e., candle and pencil) objects influenced the intra-individual consistency of the reference frame use in all four samples: The intra-individual consistency was larger for inanimate than for animate items.

Despite the interactions between sensorimotor experience, language, and culture and their impact on temporal and spatial representations, we suggest investigating further the extent to which the activation of embodied information depends on the features of the stimuli and the context [52]. For example, in a recent study, Lebois et al. [52] provided evidence that the activation of embodied information depends on its relevance for the respective tasks. Transferred to the present study: Due to the fact that the answer to the ambiguous questions can be given without considering the simultaneously executed movement, it is conceivable that movement would have modulated the reference frames if the movement had been necessary to solve the target questions (e.g., if participants would have to walk forward/backward to provide an answer). This importance of a functional connection between prime and target for embodied cognition effects could be explored in future studies.

Additionally, although movement did not modulate temporal and spatial reference frames in the study at hand in adults, one could speculate that movement might be necessary in infants for developing different reference frames. We therefore propose that a promising approach to disentangle the ‘ravel’ of the moving body and temporal and spatial representations is to look at the development of ego-moving and object-moving reference frames from early infancy onwards and to take a developmental approach to embodied cognition effects in general [53].

## Conclusion

To conclude, although further research is needed to explore the effects of whole-body movements on temporal and spatial concepts, the results of the present study seem to speak against a modulation of temporal and spatial reference frames by means of whole-body movements. Even though abstract motion (e.g., counting forward or backward) activates temporal reference frames (e.g., [18]) the present experiments indicate that this may not hold for *concrete* whole-body movement.
